# A Hitchhiker’s Guide to Supplying Enzymatic Reducing Power into Synthetic Cells

**DOI:** 10.1021/acssynbio.3c00070

**Published:** 2023-04-13

**Authors:** Michele Partipilo, Nico J. Claassens, Dirk Jan Slotboom

**Affiliations:** † Department of Biochemistry, Groningen Institute of Biomolecular Sciences & Biotechnology, University of Groningen, Nijenborgh 4, 9747 AG Groningen, The Netherlands; ‡ Laboratory of Microbiology, Wageningen University, Stippeneng 4, 6708 WE Wageningen, The Netherlands

**Keywords:** redox reactions, nicotinamide adenine dinucleotides, minimal metabolism, synthetic cells, vesicles, electron donors, dehydrogenases

## Abstract

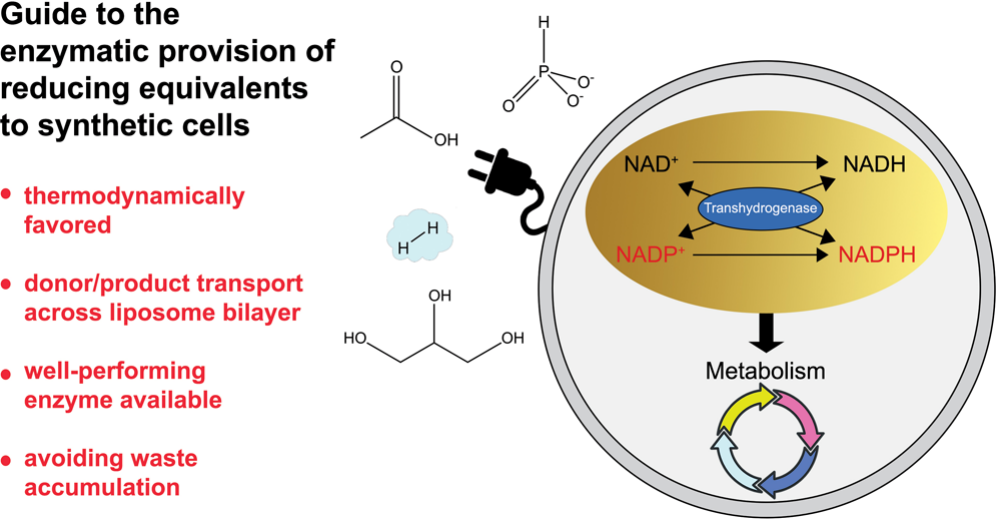

The construction from scratch of synthetic cells by assembling molecular building blocks is unquestionably an ambitious goal from a scientific and technological point of view. To realize functional life-like systems, minimal enzymatic modules are required to sustain the processes underlying the out-of-equilibrium thermodynamic status hallmarking life, including the essential supply of energy in the form of electrons. The nicotinamide cofactors NAD(H) and NADP(H) are the main electron carriers fueling reductive redox reactions of the metabolic network of living cells. One way to ensure the continuous availability of reduced nicotinamide cofactors in a synthetic cell is to build a minimal enzymatic module that can oxidize an external electron donor and reduce NAD(P)^+^. In the diverse world of metabolism there is a plethora of potential electron donors and enzymes known from living organisms to provide reducing power to NAD(P)^+^ coenzymes. This perspective proposes guidelines to enable the reduction of nicotinamide cofactors enclosed in phospholipid vesicles, while avoiding high burdens of or cross-talk with other encapsulated metabolic modules. By determining key requirements, such as the feasibility of the reaction and transport of the electron donor into the cell-like compartment, we select a shortlist of potentially suitable electron donors. We review the most convenient proteins for the use of these reducing agents, highlighting their main biochemical and structural features. Noting that specificity toward either NAD(H) or NADP(H) imposes a limitation common to most of the analyzed enzymes, we discuss the need for specific enzymes—transhydrogenases—to overcome this potential bottleneck.

## Introduction

Since the early 1990s, when *in vitro* physicochemical conditions leading to synthetic self-replicating lipid vesicles (liposomes) were initially explored,^1,2^ efforts toward the construction of artificial cells by rationally putting together well-defined (bio)chemicals have been intensified. The encapsulation into liposomes of more sophisticated biomolecular mixtures, such as the translational machinery,^3,4^ set a next milestone to recreate “bottom-up” biomimetic systems with greater functional complexity. In recent years, the growing focus on synthetic cells assembled by a bottom-up approach led to the exciting development of new subfields within a broader research area defined as synthetic biology.^5−9^ One of these fields covers the design of enzymatic pathways/modules able to perform metabolic functions typical of living systems within the compartment of synthetic liposomes. Central energy converting modules have been successfully reproduced inside liposomes, including the production of ATP,^10−12^ the generation of light-driven proton motive force,^13^ and the reduction of NAD(P)^+^.^14^ Nonetheless, the delineation of an essential metabolism that guarantees physicochemical homeostasis, growth, and replication still remains a daunting task, because the metabolic modules must operate in a coordinated way. Therefore, early design choices of a synthetic metabolism are crucial to allow for expansion and combination of modules. The amount of possible choices is large, because life has found different solutions to thrive in the most disparate environments and variable access to nutrients thanks to a wide range of genes coding for enzymes that allow it to harness the available resources.^15,16^

**Figure 1:**
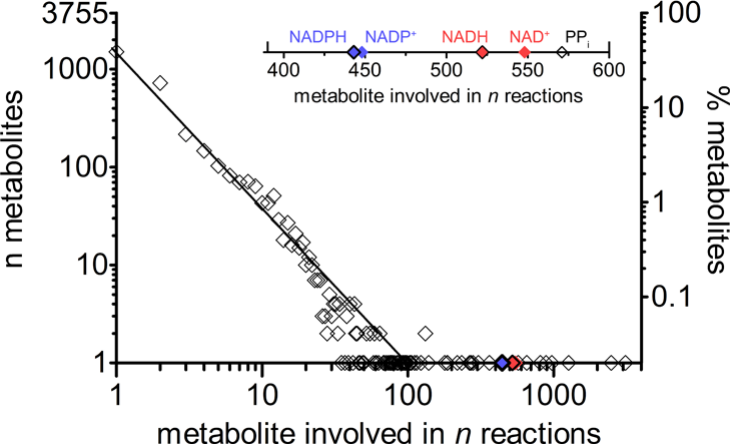
NAD(H) and NADP(H) occurrence among the enzymatic reactions of *Escherichia coli*. Metabolites involved in cellular reactions tend to follow a power-law distribution (generically described by the equation *y* = *C*/*x*^*a*^), in which the majority of the metabolites is involved in a small number of reactions while a small group of metabolites takes part in up to 100 enzyme-mediated reactions. On the left *y*-axis is reported the absolute number of metabolites (*n*), and on the right *y*-axis is shown the same number of metabolites in terms of percentage (%). The diagonal approximates the linearity of the power-law distribution in a double logarithmic scale graph, as also shown by Schmidt et al.^17^ The restricted group of metabolites involved in more than a hundred reactions represent exceptions to the power-law distribution, and are called hub metabolites. The upper right inset highlights the number of reactions dependent on NAD^+^ (red diamond), NADH (red-black diamond), NADP^+^ (blue diamond), and NADPH (blue-black diamond). The data set was extracted from the *Escherichia coli* Metabolome Database (http://www.ecmdb.ca).

Despite the enormous diversity of metabolic pathways, there are a few molecules (hub metabolites) taking part in hundreds of different reactions in all the living systems.^17,18^ Specifically, the nicotinamide adenine dinucleotides NAD(H) and NADP(H) are involved in approximately 2000 out of almost 8000 reactions annotated in the *Escherichia coli* Metabolome Database^19^ (Figure 1), and therefore designated as hub metabolites. Their main function is to transfer reducing equivalents to or from a plethora of reactants, via the catalytic action of specific oxidoreductases.^20^ The existence of two nicotinamide cofactors that differ only in structure at the position 2′ of the riboside ring where NAD(H) has a hydroxyl group, which in NADP(H) is phosphorylated, allows for the distinction of pathways that break down (catabolism) or assemble (anabolism) molecular building blocks.^21^ NADPH is mostly involved in anabolic reduction reactions and NAD^+^ in catabolic oxidations. For instance, the cellular NADPH concentration in most bacteria is usually kept much larger than the NADP^+^ concentration, while the NAD^+^/NADH ratio is lower, thus enabling oxidations.^22^ However, this dichotomy is not a strict rule for all living systems, and the use of NADP(H) or NAD(H) in specific reactions is dependent on enzyme specificities, thermodynamic constraints, and cellular conditions.^22−24^ The continuous reduction and reoxidation of the nicotinamide pools is necessary to feed further enzymatic reactions involved in the biosynthesis of crucial biomolecules such as sugars, nucleotides, lipids, and cofactors.^20,22,25,26^

Several reviews have discussed the enzymatic regeneration of NAD((P)H) cofactors both *in vivo* and *in vitro*, principally for (bio)manufacturing purposes.^22,27−30^ However, building a redox regeneration pathway that supports a minimal metabolic network enclosed in liposome confinement and built in a bottom-up manner poses specific challenges, which we will address in this work. We review (i) the choice of the suitable electron donor (substrate) for reduction of nicotinamides from a thermodynamic perspective, (ii) transport modes of the potential electron donating substrates across the phospholipid bilayer, (iii) the availability of NAD(P)^+^-dependent enzymes that use the substrate, (iv) the fate of the reaction product formed during the nicotinamide reduction, which preferentially should not accumulate in the lumen of the synthetic cells, and (v) the compatibility of the redox power provision with an aerobic environment, considering the integration of the redox module in a more complex metabolic network that might benefit from the presence of oxygen. These apparently simple conditions narrow the number of compounds to take into account as convenient electron donors, and enzymes to be implemented for utilization of the reducing power. We focus here on electron supply by chemical donor molecules rather than photoinducible water-splitting systems (e.g., photosystems I and II).^31^ While photosynthetic reactions are intriguing options to regenerate NAD(P)H, the reconstitution of the full photosystem and accessory proteins to achieve a functional redox module demands a large number of proteins and coenzymes. Additionally, this would tie the electron supply to light exposure of the synthetic cell for operation.

Finally, we discuss enzymatic transhydrogenation, which is of relevance to synthetic cells because dehydrogenases oxidizing the respective electron donating substrate usually exhibit cofactor specificity for either NAD(H) or NADP(H), whereas a synthetic metabolism would require both the distinct cofactors in certain ratios for energy production and biosynthetic purposes, respectively.

## The Rationale for Selecting Electron Donors for Nicotinamide Cofactors in Synthetic Cells

### 1 The Thermodynamics

**Figure 2:**
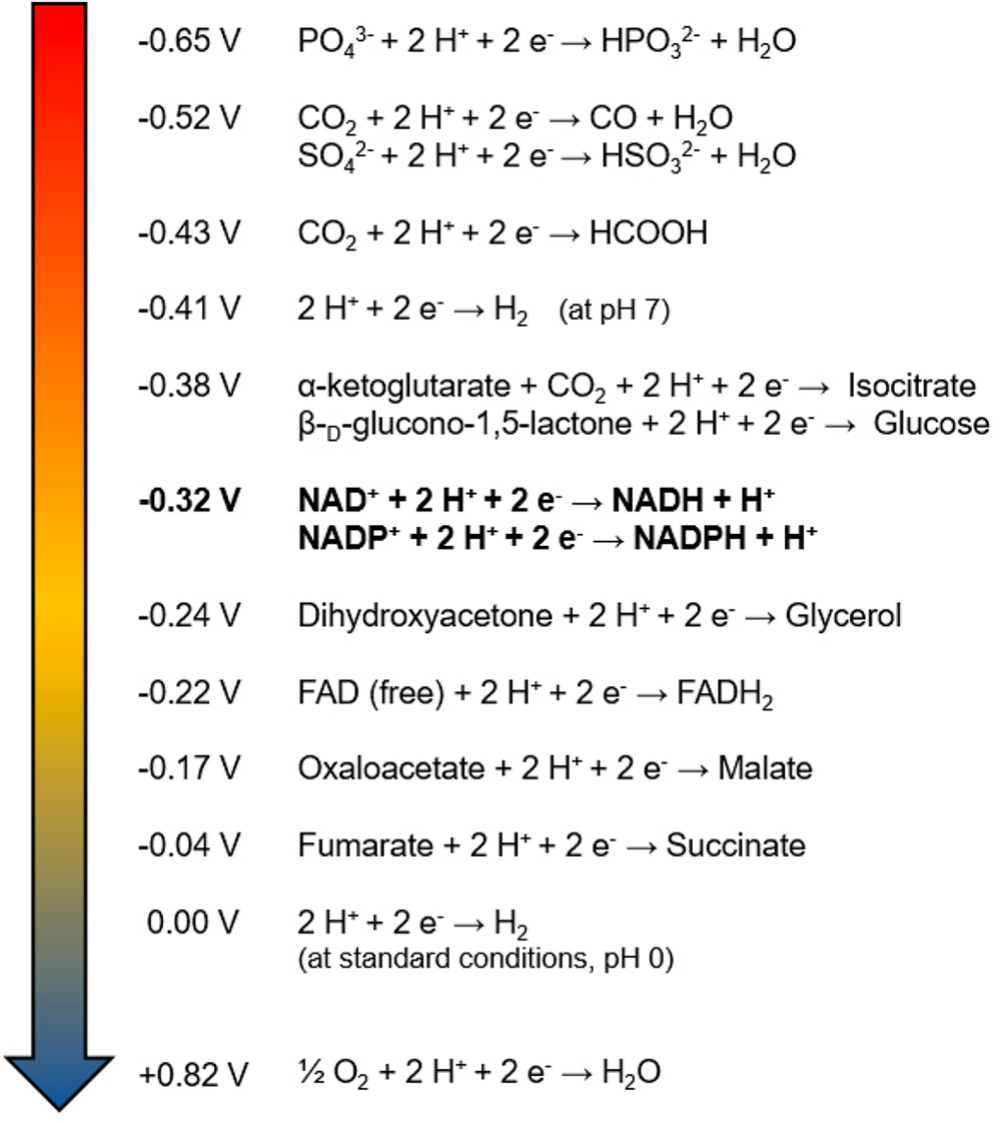
Overview of the standard reduction potentials (*E*°′, at 25 °C, pH 7.0) of the half-reactions of candidate electron donors.^32−36^

Thermodynamic feasibility is an essential factor in the design of metabolic pathways: cell-free synthetic pathways must rely on initial thermodynamic analysis to ensure the desired directionality of metabolic fluxes.^37,38^ When constructing synthetic cells, the same principle must be applied in linking the different pathways for synergic functionality of the overall enzymatic network. For redox reactions, the spontaneity of a reaction under biological standard conditions (Δ*G*°′ < 0, pH 7.0, 25 °C, 1 atm) can be calculated from the difference in reduction potentials (Δ*E*°′) between the reactants, according to eq 1:
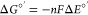
1where Δ*G*°′ stands for the change in Gibbs free energy under biological standard conditions, *n* corresponds to the number of electrons involved in the reaction, and *F* is the Faraday constant. Since the electrons spontaneously move in the direction of the redox pair with the most positive *E*°′ value, the hydride transfer from a reduced to an oxidized species is constrained by the reduction potentials of the involved reactants. A backward electron flow can occur by shifting the relative concentrations of reactants, but it may require substantial concentration differences to overcome an unfavorable equilibrium.^39^ Taking pH 7.0 for a synthetic cell to operate, the standard reduction potential value *E*°′ of the half-reaction NAD(P)^+^/NAD(P)H at −0.32 V limits the number of potential electron donors. With their lower *E*°′ values (Figure 2), the phosphite/phosphate, sulfite/sulfate, carbon monoxide (CO)/CO_2_, d-glucose/CO_2_, formic acid/CO_2_, molecular hydrogen (H_2_)/H^+^, and isocitrate/α-ketoglutarate redox couples emerge as thermodynamically favorable “reducing agents” for nicotinamides.

The malate/oxaloacetate pair (with *E*°′ value of −0.17 V) represents a less favorable option from a thermodynamic perspective. Under standard conditions, the electrons would move from NAD(P)H to oxaloacetate, with an equilibrium constant strongly shifted toward the formation of NAD^+^ and malate (*K*_eq_ ≈ 3 × 10^5^).^40^ However, with elevated concentrations of malate the reaction can also lead to reduction of NAD(P)^+^, as it happens *in vivo* in the tricarboxylic acid cycle.^41^

The succinate/fumarate redox pair has an *E*°′ value (−0.04 V) even further above NAD(P)H/NAD(P)^+^ making it virtually impossible to reduce NAD(P)^+^ with electrons from succinate. In nature also no NAD(P) ^+^-dependent succinate dehydrogenase is known, and in living cells this reaction operates with quinones as (high potential) electron acceptors.

### 2 The Accessibility to a Membrane-Defined Compartment Characterized by Selective Permeability

In a cell-mimicking system, the phospholipid membrane delineates a selectively open system.^42^ The NAD(P)^+^ cofactors and the enzymes remain confined within the lumen of the liposomal compartment once encapsulated due to their membrane-impermeable nature. Only small uncharged molecules can pass through the membrane unassisted, while charged and larger molecules require specific membrane proteins to allow the transport of solutes.^33^ While pore-forming proteins (e.g., cytolysin A, α-hemolysin, fragaceatoxin C, OmpF, etc.)^43^ can be employed to enable the passage of multiple reactants within a certain size depending on the cutoff of the specific pore, most membrane transporters are characterized by substrate-specificity^44^ that ensures a high degree of selectivity and control of membrane-impermeable reactants entering or leaving the liposomal lumen. Although pore-forming proteins have found some useful applications in cell-like systems,^45−47^ they are not ideal for sustaining the out of equilibrium status over the long-term, hindering the retention and internal recycling of the small molecules necessary for metabolic homeostasis.^42^ We therefore refer specifically to transporters as membrane proteins of choice in bottom-up synthetic cells to mediate the translocation of membrane-impermeable solutes.

When an external electron donating substrate is membrane-impermeable, the addition to the system of a transporter is needed in order to ensure the supply of substrate to the luminal dehydrogenase: this is the case when utilizing electron donors such as phosphite, malate, isocitrate, and d-glucose. In contrast, small molecules such as H_2_, formic acid and glycerol can permeate across the membrane by unassisted diffusion. For formate, at physiological pH, the anionic form (HCOO^–^) prevails over the protonated formic acid (p*K*_a_ = 3.75). Only the latter species is membrane permeable but the acid–base equilibrium does not limit diffusion through phospholipid membranes: its permeability coefficient has been estimated at 719 × 10^–5^ cm/s in 1,2-dioleoyl-*sn*-glycero-3-phosphocholine (DOPC) vesicles.^48^ Although almost 2 orders of magnitude less permeable than formic acid,^48^ glycerol is also membrane-permeable and consequently does not strictly require a specialized transporter. Glycerol has being recognized in recent years as an attractive hydrogen donor for the synthesis of value-added chemicals.^49,50^

Finally, the electrochemical gradient resulting from the import of an electron donor must be taken into consideration because it may affect the pH gradient across the membrane, cause osmotic changes, and may lead to variations to the electrical potential difference across the membrane, if net charge translocation takes place, which could alter the functionality of the synthetic cell.^51^ Potentially, a careful design of the phenomena that take place at the level of the membrane enclosing the compartment (e.g., proton gradient, membrane potential) can be exploited for other essential processes, such as energy production^52^ or cell division.^53^ The same reasoning applies to products that diffuse out passively or that are exported by protein-mediated action.

### 3 The Availability of Annotated NAD(P)^+^-Dependent Dehydrogenases

A low reduction potential and accessibility to the lumen alone are not sufficient to select suitable hydride donors for NAD(P)^+^ coenzymes. There must also be oxidoreductases mediating the oxidation of the electron donor in favor of the specific reduction of the nicotinamide cofactor. The absence of a catalyst would make the time scale of the reaction incompatible with life-like systems.^54^ Two clear examples of such limitation are represented by the redox couples sulfite/sulfate and carbon dioxide/carbon monoxide. Although SO_4_^2–^/HSO_3_^2–^ has a much more negative reduction potential (*E*°′ = −0.52 V)^32^ than the nicotinamide couples, there is not a single known enzyme that transfers reducing equivalents directly from sulfite to NAD^+^ or NADP^+^. Sulfite can be oxidized enzymatically only by reducing either ferricytochrome *c* by sulfite dehydrogenase (EC 1.8.2.1) in bacteria or oxygen by the action of sulfite oxidase (EC 1.8.3.1) in plants and animals.^55^ Similarly, CO would constitute a suitable electron donor for nicotinamides from a thermodynamic point of view (CO_2_/CO, *E*°′ = −0.52 V), yet carbon monoxide dehydrogenases (CODHs) mostly use oxidized ferredoxin (EC 1.2.7.4)^56^ or quinones (EC 1.2.5.3)^57^ as reaction partners. To date, the oxygen-tolerant CODH from the hyperthermophilic archaeon *Aeropyrum pernix* has been the only purified homologue reported to reduce NAD(P)^+^ cofactors.^58^

Contrary to sulfite and carbon monoxide, glycerol can be oxidized by the action of specific NAD^+^-dependent glycerol dehydrogenases (EC 1.1.1.6)^59^ forming dihydroxyacetone and NADH. The thermodynamic bottleneck between the redox couples dihydroxyacetone/glycerol (*E*°′ = −0.24 V)^36^ and NAD(P)^+^/NAD(P)H is smaller than what was mentioned above for the dicarboxylates, and therefore is overcome by relatively minor changes in concentration of the reactants. These factors led us to consider glycerol as a suitable electron donating substrate for nicotinamide cofactors.

For the later selected electron donors, NAD(P)^+^-dependent dehydrogenases are available as extensively discussed below.

### 4 NAD((P)H) Regeneration Systems without Undesired or Reaction Products

**Figure 3:**
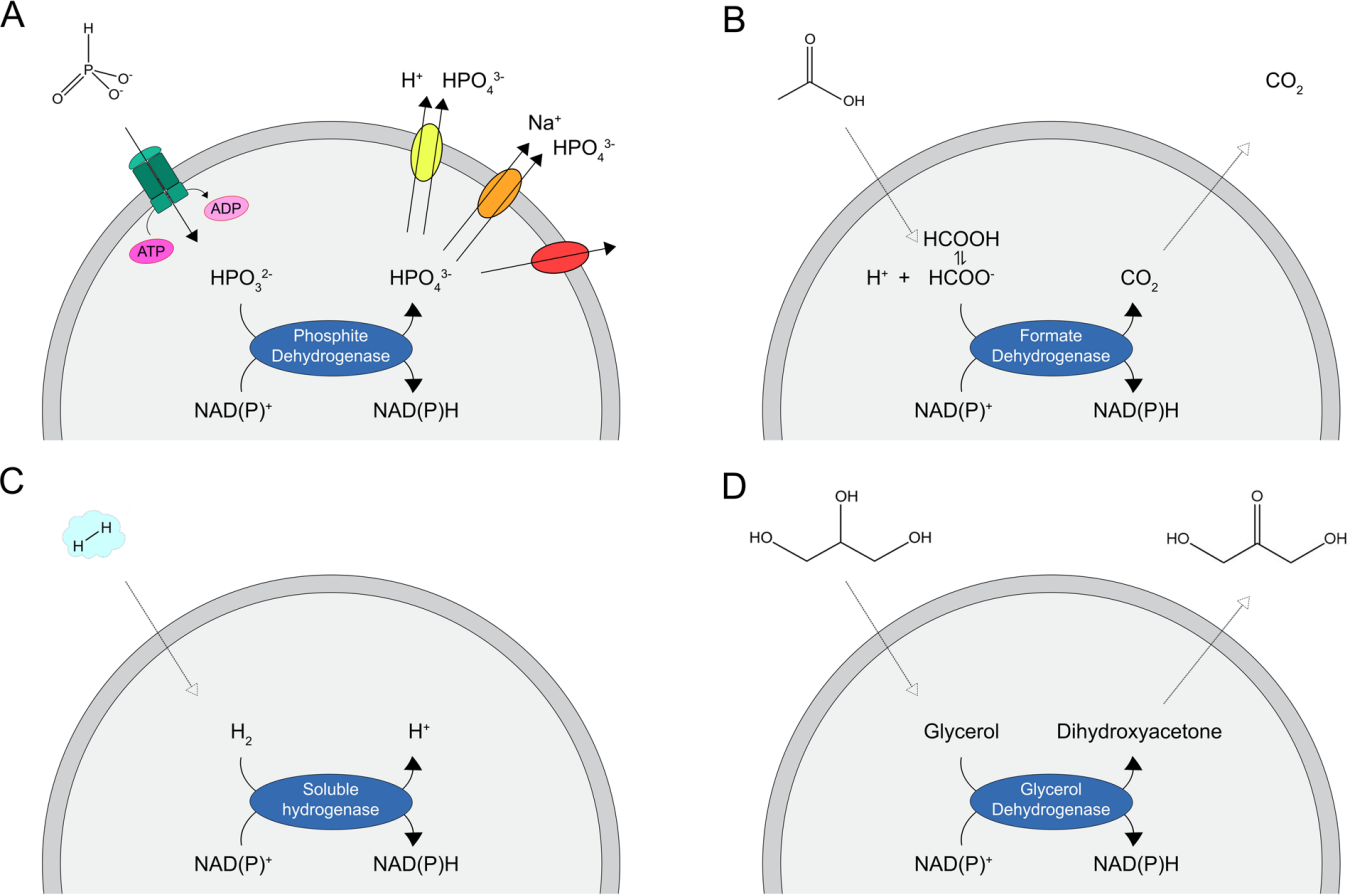
Strategies for the orthogonal provision of reducing power in synthetic cells. Both the electron donors for the nicotinamide coenzymes and the respective reaction products have two possible ways to move across the membrane: by protein-mediated action (A) or by unassisted permeation (B–D). (A) Phosphite is internalized by an ATP-binding cassette (ABC) transporter (shown in green), while its reaction product phosphate can be exported in symport with a proton (in yellow) or a sodium ion (in orange), or by facilitated-diffusion (in red). (B–D) Formate, molecular hydrogen and glycerol, as well as carbon dioxide and dihydroxyacetone freely and readily permeate the phospholipid bilayer. In order to transfer the hydrides from the donor to the NAD(P)^+^ cofactors, the cell-like compartments need to be equipped with specific dehydrogenase(s).

Synthetic cells must perform a diverse set of biochemical reactions to successfully maintain their homeostasis and eventually be able to reproduce themselves. Ideally, any new reactant supplied from the outside has to fulfill specific functions by undergoing a catalytic conversion that results in the formation of new building blocks and/or enables the luminal regeneration of needed biomolecules. Therefore, when the reaction products have no use and can be even detrimental to the metabolic network, it is necessary to remove them from the liposome compartment to avoid accumulation preventing any possible interference with other metabolic components. To efficiently ensure the removal of a specific membrane-impermeable product, it may be possible to take advantage of a membrane protein antiporter exporting the undesired reaction product in exchange with an external substrate of interest, as showcased in liposomes encapsulating the arginine breakdown pathway for ATP production:^10^ in this case, the product ornithine is exported out in exchange with arginine, that is the initial substrate required to trigger the pathway activity. With regard to redox reactions, the best electron donors are the compounds whose oxidation transfers hydrides to NAD(P)^+^ cofactors while the coproducts can be easily exported out (Figure 3). This criterion of “orthogonality” leads to the exclusion of some of the above-mentioned compounds that thermodynamically are very advantageous with their low *E*°′ value, for instance d-glucose and α-ketoglutarate, as their direct oxidation products, β-d-glucono-1,5-lactone and alpha-ketoglutarate, cannot be easily exported. Even though both molecules could partly serve as building blocks, this would be challenging to stoichiometrically balance with the need for reducing equivalents in a synthetic cell, and likely still some transport mechanisms would be needed to export a part of this product pool.

Another potential electron donor substrate is methanol, however its oxidation product formaldehyde is highly toxic and known to cross-link proteins and DNA and hence is an undesired option.^60^

To further elaborate on the export issue for d-glucose products, this sugar can, apart from single-step oxidation to β-d-glucono-1,5-lactone, also be fully oxidized to the easy-to-export product CO_2_. In living systems the full oxidation is performed using multienzymatic metabolic pathways (Emden–Meyerhof or Entner–Doudoroff glycolysis in combination with the citric acid cycle).^61^ These multistep pathways are obviously extremely advantageous *in vivo*, but they are challenging to use in the context of a synthetic cell, as they would require a considerable number of enzymes to be purified and reconstituted, which may lead to technical complications as well as potential regulatory complexity. In addition, the tricarboxylic acid cycle not only generates NAD(P)H but also reduced quinol, which is not necessarily desired and requires another regeneration system such as a respiratory chain, further increasing complexity and hindering orthogonality of the redox regeneration system.

As an alternative for the glycolytic routes using d-glucose, a single enzyme such as d-glucose dehydrogenase (GDH, EC 1.1.1.47) could be employed to directly form NAD(P)H while oxidizing the hexose carbohydrate into β-d-glucono-1,5-lactone. Nonetheless, export of β-d-glucono-1,5-lactone is not known to occur via an antiporter with glucose, nor any other form of transport or minimal metabolic route for disposal is known for this product, and therefore accumulation of the metabolite would occur.

The same applies for the isocitrate/α-ketoglutarate couple, unless a specific carboxylate antiporter can be identified and successfully utilized in the desired direction (uptake of isocitrate and export α-ketoglutarate), which would allow to retain only the reducing equivalents, as NAD(P)H, in the lumen of synthetic cells enclosing an isocitrate dehydrogenase (EC 1.1.1.41 and 1.1.1.42), since the carbon dioxide produced together with α-ketoglutarate would diffuse out.

### 5 The Preference for Aerobic Pathways by Envisioning Metabolic Complexity

Some enzymes catalyzing redox reactions are oxygen-sensitive, and only functional in anaerobic conditions (e.g., many oxidoreductases and respiratory enzymes from anaerobic microorganisms^62−64^) forcing the assembly and function of the vesicles to an inconvenient oxygen-free environment. Indeed, it is technically challenging and practically undesirable to prevent the presence of oxygen in each stage of the liposomal preparation, starting from the biochemical encapsulation to the functional assays of vesicle activity, regardless of the chosen technique or the size of the phospholipid compartments.^65^ On top of this technical limitation, it may not be desirable to exclude molecular oxygen, as it drives one of the most efficient energy conversion processes as a strong oxidizing agent, that is the generation of a proton motive force across the membrane, which subsequently is used for the synthesis of ATP.^66,67^ Considering the high demand for ATP calculated to support the major functions in a synthetic cell,^11^ the option of a minimal respiratory system with oxygen as final electron acceptor is ultimately to be considered: it has been estimated that aerobic respiration in microbes leads to an ATP yield per electron that is 1–2 orders of magnitude higher than alternative anaerobic processes (e.g., denitrification, sulfate and ferric respiration, methanogenesis).^68^ In this respect, the use of complex I concomitantly with ubiquinone and an alternative oxidase has been proved as a promising strategy in (proteo)liposomes to fuel a minimal respiratory chain with oxygen as a final electron sink.^69^ Its recent coupling with an ATP-synthase^70^ has even shown the potential of generating ATP by taking advantage of the proton motive force established by the proton-pumping action of the mitochondrial complex I; however, in this case ATP was produced outside the vesicles as a result of the preferential orientation of complex I.

Furthermore, artificial cells capable of transcription and translation (TX-TL) are often tested and optimized with green fluorescent proteins and derivatives to monitor the protein synthesis efficiency. Folding and maturation of these fluorescent proteins is strictly dependent on dioxygen availability.^3^ Hence, the enzymes further discussed in this review are oxygen tolerant.

## The Protein Toolbox for Redox Reactions Inside Synthetic Cells

The aforementioned considerations led us to identify four main candidate compounds as viable electron donors for nicotinamides within artificial cells (Table 1): phosphite, formate, molecular hydrogen, and glycerol. We now proceed to describe the most efficient ways exploit their reducing power (Figure 3) by focusing on the enzymes that oxidize them (dehydrogenases listed in Table 2) and, in the case of phosphite/phosphate, allow their transport through the lipid bilayer (membrane transporters). The other donors and products can passively enter and leave the cell.

**Table 1:** Properties of the Electron Donating Substrates and Respective Reaction Products Selected as Suitable for Synthetic Cells^a^

compound	PubChem ID	MW	standard reduction potential (V)	solubility	transport mode	*P* × 10^–5^ (cm/s)
Phosphite (HPO_3_^2–^)	107908	78.97		high	primary active	impermeable
Phosphate (PO_4_^3–^)	1061	94.97	–0.65	high	secondary active	impermeable
					facilitated diffusion	
Formate (HCOO^–^)	283	45.02		high	passive diffusion	719
Carbon dioxide (CO_2_)	280	44.01	–0.43	low	passive diffusion	1 × 10^5^
Hydrogen (H_2_)	783	2.02		low	passive diffusion	N.D.
Hydrogen ion (H^+^)	1038	1.01	–0.41	high	primary active	1 × 10^–9^
					secondary active	
Glycerol (C_3_H_8_O_3_)	753	92.09		high	passive diffusion	2
Dihydroxyacetone (C_3_H_8_O_3_)	670	90.08	–0.24	high	passive diffusion	N.D.

a The reported permeability coefficients (*P*) refer to (DOPC) vesicles.^48,71^ N.D. stands for “not determined” in an experimental setup.

**Table 2:** Features of the NAD(P)^+^-Dependent Dehydrogenases Oxidizing Suitable Electron Donors for Nicotinamide Coenzymes

enzyme name	enzyme code	organism	substrates	*K*_M_ (mM)	*k*_cat_ (s^–1^)	molar mass (kDa)	oligomeric state	pH optimum	temp. optimum (°C)	ref.
Phosphite dehydrogenase	1.20.1.1	*P. stutzeri*	NAD^+^	0.05	3.2	70	homodimer	7.0–8.0	35	(34,72)
			phosphite	0.05						
Formate dehydrogenase	1.17.1.9	*P. species* 101	NAD^+^	0.08	7.5	90	homodimer	6.0–9.0	63	(73,74)
			formate	15.0						
Soluble [NiFe]-hydrogenase	1.12.1.2	*C. necator*	NAD^+^	0.20	109.0	170	heterotetramer	8.0	35	(75−77)
			H_2_	0.04	143.0	210	heterohexamer			
Glycerol dehydrogenase	1.1.1.6	*G. stearothermophilus*	NAD^+^	0.52	7.4	320	homo octamer	9.0–10.0	–	(59,78)
			glycerol	50.0						

## Phosphite

**Figure 4:**
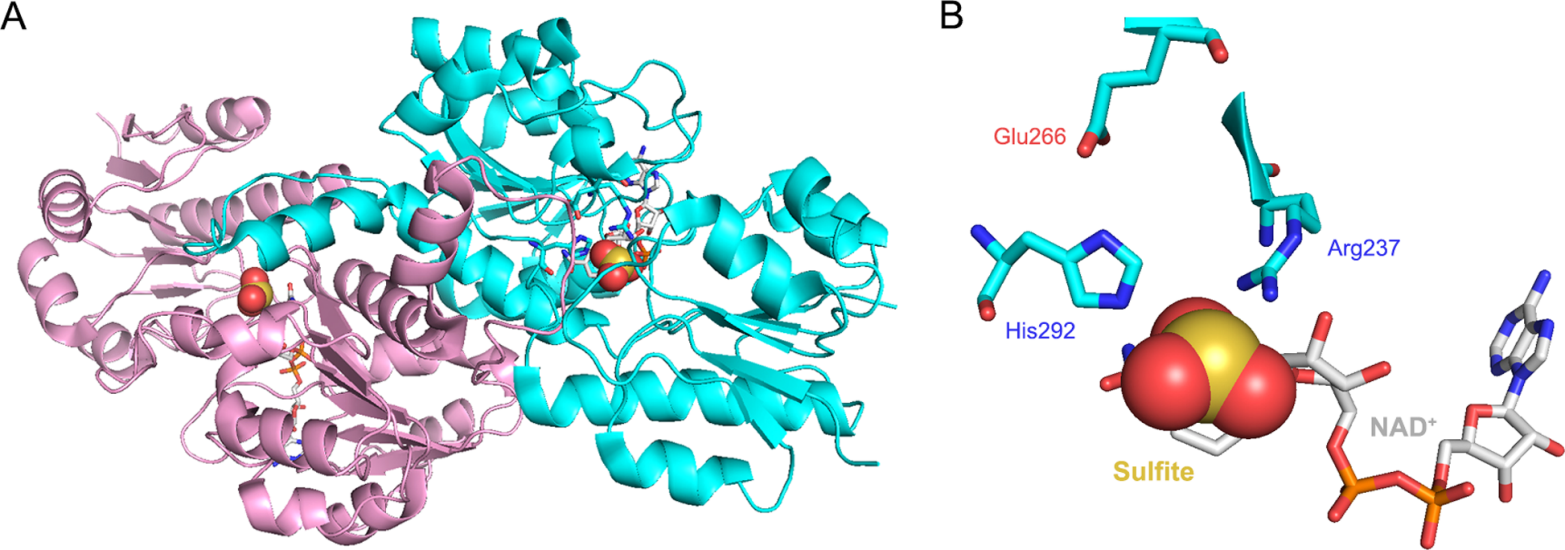
Phosphite dehydrogenase from *P. stutzeri* WM 88. (A) The homodimeric assembly of the holoenzyme in complex with NAD^+^ and sulfite, specific inhibitor of PDH (PDB entry: 4E5K). (B) A zoom into the PDH active site. The conserved catalytic triad Arg237, Glu266, and His292 allows catalysis with a proposed acid–base mechanism. The nucleophilic attack on the phosphite (here replaced by the competitive inhibitor sulfite) is initiated by histidine—via a water molecule—and is oriented by the combined action of arginine and glutamate, resulting in the reduction of the nicotinamide coenzyme.

Phosphite is cheap and has been previously advocated as a suitable compound for the regeneration of nicotinamide cofactors *in vitro*.^28^ Thermodynamically, the PO_4_^3–^/HPO_3_^2–^ pair (*E*°′ = −0.65 V) is well below that of the NAD(P)^+^/NAD(P)H couple, resulting in an equilibrium constant (*K*_eq_ ≈ 10^11^) strongly shifted toward the cofactor reduction at pH 7.0.^34^ The redox reaction is catalyzed by phosphite dehydrogenase (PDH, EC 1.20.1.1), a homodimeric NAD^+^-dependent oxidoreductase (Figure 4A) well characterized from *Pseudomonas stutzeri* WM 88.^72^ PDH is structurally related to the family of the D-2-hydroxyacid dehydrogenases, with which it shares the catalytic triad Arg-Glu-His in the active site for the hydride transfer (Figure 4B).^79^ Although PDH shows a clear preference for NAD^+^ over NADP^+^ with 100 times higher catalytic efficiency (*k*_cat_/*K*_M_) for the coenzyme without the phosphate at the 2′ position, the cofactor specificity can be broadened through a double mutation (E175A/A176R),^80^ allowing the reduction of both cofactors with a comparable *k*_cat_/*K*_M_, but slightly in favor of NADP^+^. The wild-type PDH has micromolar range affinity for both the native substrates, working efficiently in a pH range between 7.0 and 8.0 and below 40 °C (Table 2). The PDH activity is suppressed in the presence of sulfite in a similar concentration range (*K*_i_ = 16 μM) through a mechanism of competitive inhibition with the phosphite ion. The ionic strength of the reaction buffer is a crucial parameter to keep under control for optimal PDH activity, as suggested by the enzymatic inactivation observed by increasing the concentration of electrolytes such as sodium chloride.^72^

While there is in-depth biochemical and structural insight of the enzyme utilizing phosphite, knowledge of the membrane proteins that mediate its transport is still fragmentary. PtxABC and PhnCDE are the main transporters known to date that import phosphite. They have been identified from microorganisms capable of growing on the compound as the only source of phosphorus, as *P. stutzeri* WM 88,^81^*Trichodesmium erythraeum* IMS101,^82^ and *Prochlorococcus marinus*.^83^ Both PtxABC and PhnCDE are ATP-binding cassette (ABC) transporters composed of two nucleotide-binding subunits (PtxA or PhnC), two transmembrane subunits (PtxC or PhnE), and a solute-binding protein (PtxB or PhnD). The purified solute binding proteins have high affinities for phosphite with dissociation constant (*K*_D_) values in the nanomolar range. These proteins bind phosphate with much lower affinity (2–4 orders of magnitude higher *K*_D_ depending on the specific protein).^84^ Their crystallographic structures have elucidated the essential amino acids involved in the substrate recognition. In contrast, no functional information on either the ATP binding domains or the transmembrane domains of these ABC transporters is yet available *in vitro*, although the predicted structures of PtxA and PtxC from *P. stutzeri* are accessible in the AlphaFold database (AF-O69051-F1 and AF-O69053-F1, respectively).^85^

In view of the construction of a synthetic cell, the successful reconstitution of one among these phosphite transporters and the consequent use of PDH would not only provide reducing power by enabling the phosphite-consuming reaction, but it would also produce the phosphate ion. Phosphate is a ubiquitous osmolyte in living systems and an integral part of the main biomolecules (nucleic acids, proteins, lipids, carbohydrates). The design of a metabolic circuit that would couple the redox gain from phosphite oxidation with a phosphate sink (nucleosides, inositols, etc.)^86^ would maximize the potential yield from both products of the PDH-mediated reaction, preventing the accumulation of an otherwise dead-end metabolite. However, likely the demand for phosphate is lower than its production. This would require export of phosphate for which different transporters are available. We will not elaborate here on phosphite/phosphate antiport—which would be a simple and elegant solution—as there is only a single membrane protein (PtdC from *Desulfotignum phosphitoxidans*)^87^ which has been proposed as a phosphite/phosphate transporter, but no experimental evidence, neither *in vivo* nor *in vitro*, has been provided to date in support of this function.

There are a few alternatives based on known membrane transporters that can be implemented to remove the phosphate ion from the vesicular compartment (Figure 3A): first, the export of inorganic phosphate in combination with the removal of a proton (PitA from *E. coli*,^88^ TCDB 2.A.20.1.1; PiPT from *Piriformospora indica*,^89^ TCDB 2.A.1.9.10; PIC from pig mitochondria,^90^ TCDB 2.A.29.4.2); second, the symport of phosphate and sodium ion out of the lumen (YjbB from *E. coli*,^91^ TCDB 2.A.58.2.1); third, the facilitated diffusion of inorganic phosphate alone (Pho1 from *Arabidopsis thaliana*,^92^ TCDB 2.A.94.1.1). With the exception of the purified and biochemically active PIC^90,93^ and the crystallized PiPT,^89^ all the other mentioned candidate transporters have not been characterized *in vitro*, but only partially *in vivo*. Considering the nontrivial troubleshooting involving the overproduction, purification and functional characterization (lipid and detergent requirements, membrane orientation, activity assays, etc.)^94−96^ of membrane proteins, the choice of the phosphite/phosphate pair could require substantial optimization work. Thus, the biochemical and structural gap constitutes currently one of the major limitations to be addressed in order to apply phosphite and phosphate transporters in bottom-up systems such as artificial cells. In the absence of a functionally characterized phosphite/phosphate antiporter, this redox couple would depend on ATP for the phosphite import via the ABC-transporters; typically a synthetic cell would be equipped with an ATP-regeneration module, but this redox system would increase the demand from that module.

## Formate

While formate in high concentrations can be toxic to some living organisms,^97,98^ it represents a harmless and highly soluble C_1_ compound to readily provide reducing power to artificial cells designed in a bottom-up fashion. Since it does not require a membrane transporter to cross phospholipid bilayers in the millisecond time scale,^99^ formate provides the advantage of requiring exclusively a NAD(P)^+^-dependent formate dehydrogenase (FDH) to reduce the nicotinamide cofactor, while CO_2_ is formed as a reaction product. NAD(P)^+^-dependent FDHs (EC 1.17.1.9) can be subdivided in metal-containing FDHs and nonmetal FDHs.^100^ Metal-containing FDHs are heteromeric complexes displaying a huge diversity in the overall structure (number of subunits, amount of embedded iron–sulfur clusters, presence of flavin mononucleotide as prosthetic group), but a high degree of conservation in correspondence of the active site embedding molybdenum (Mo) or tungsten (W).^100,101^ Regardless of the metal nature, Mo/W is always coordinated by two molybdopterin cofactors, a (seleno)cysteine and a terminal sulfo-group. This abundance of cofactors to be kept properly embedded and in the appropriate redox state for optimal protein activity makes metal-containing FDHs challenging for straightforward purification and storage; therefore, we consider them not very suitable for synthetic bottom-up systems.

**Figure 5:**
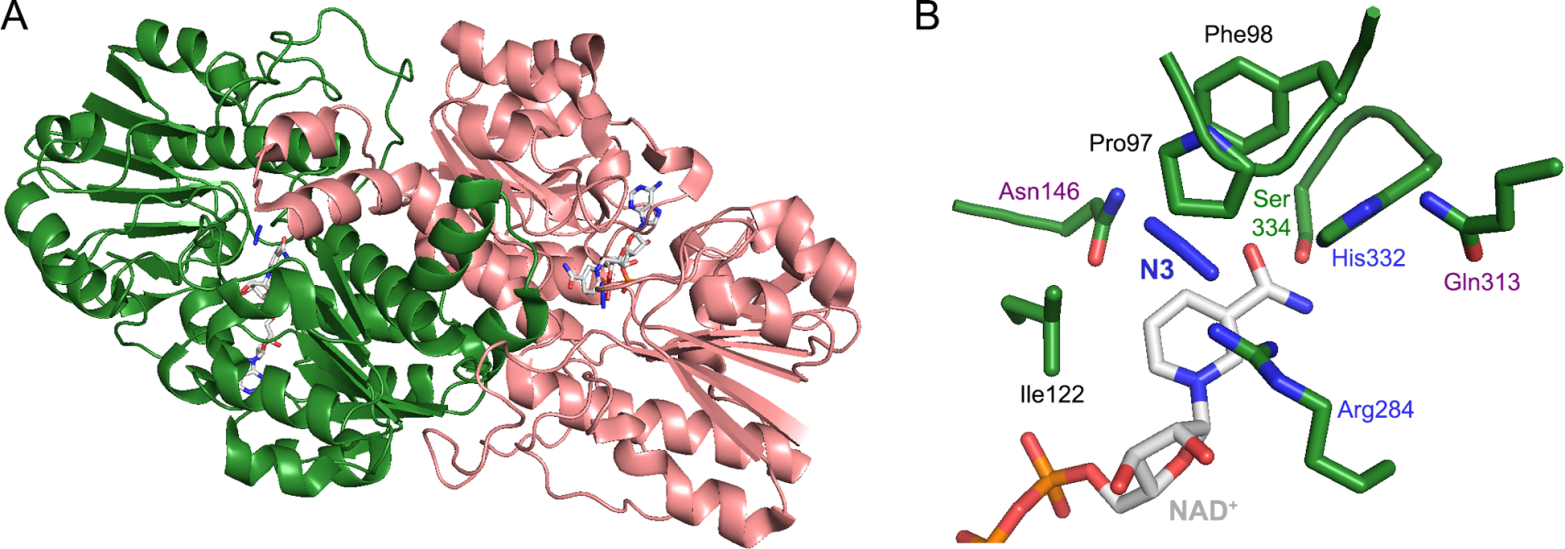
Formate dehydrogenase from *Pseudomonas* sp. 101. (A) The nonmetal containing FDH is homodimeric, here shown in complex with NAD^+^ and azide (N3), an inhibitor analogue of formate (PDB entry: 2NAD). (B) The catalysis mediated by FDH requires the involvement of multiple amino acids. His332 and Ser334 establish hydrogen bonds with the carboxamide group of the nicotinamide ring. Through a hydrogen bond with His332, Gln313 plays a key role in the FDH reaction, ensuring its characteristic wide pH optimum.^102^ The binding with formate (here replaced with the analogue, azide) is established through electrostatic interactions with the positively charged substituents of Arg284 and Asn146, while Ile122 favors the charge counterbalance of the substrate. The hydrophobic cluster (Phe97 and Pro98) promotes the spatial constriction and consequent destabilization of formate into the configuration of the reaction intermediate. Distances and other amino acids involved to a minor extent in the catalytic mechanism are here omitted, but discussed in detail somewhere else.^73,103^

On the other side, nonmetal FDHs are well characterized and easy to produce recombinantly to high purity and yield. The characterization of more than a dozen different homologues (bacterial and yeast) offers a wide selection of FDHs to draw upon to suit particular kinetic or functional requirements (e.g., chemical and thermal stability).^74^ Nonmetal FDHs are homodimers (Figure 5A) around 90 kDa with wide pH optimum, high stability above 50 °C, affinity for formate in the low millimolar range and *k*_cat_ values that do not exceed 10–15 s^–1^. Although strictly dependent on NAD^+^, their cofactor specificity can be switched with NADP^+^ with a single mutation of aspartate 221 to either alanine, glycine, serine, or glutamine (D221A/G/S/Q),^104^ as the negative charge of the aspartate which repels the phosphate group of NADP^+^ is removed. Several studies have shown that up to five additional mutations can improve the catalytic efficiency for NADP^+^, while NAD^+^ further becomes a poorer cosubstrate.^104−106^

The proposed mechanism for formate oxidation involves first the correct orientation and trapping of the nicotinamide cofactor via the concerted action of several amino acids (Figure 5B), then attacking the formate molecule through electrostatic interactions with His332 and Arg284, and then concluding the catalytic cycle by releasing the reaction products NADH and CO_2_, restoring the original conformation of the active site (thoroughly discussed in the thematic reviews^73,100^).

The use of a weak acid such as formic acid to fuel synthetic cells with reducing power would lead to luminal acidification, because it is the undissociated form (formic acid) that crosses the membrane, and then dissociates back into formate plus a proton.^99^ Nonetheless, a sufficient buffer capacity could attenuate such change in internal pH.

The enzymatic oxidation of formate fully meets the requirements for integration into a minimal compartmentalized metabolism, as the reaction product (CO_2_) diffuses out of the liposomes and only the reducing equivalents are retained within the lumen(s) in the form of NAD(P)H. We recently demonstrated the applicability of formate as a suitable electron donor using it to feed synthetic cells (both large and giant unilamellar vesicles) with reducing power via NAD(P)^+^ cofactors.^14^ The liposomal reconstitution of a nonmetal formate dehydrogenase enabled the activity of a minimal enzymatic pathway transferring hydrides to an electron sink of biological relevance for its antioxidant function, namely glutathione.

## Molecular Hydrogen

Unlike the other electron donor candidates described so far, H_2_ is highly insoluble. Although an estimate of the *P* value for dihydrogen has not yet been reported for phospholipid membranes, it is reasonable to assume it is in the range as for other small gaseous molecules such as O_2_, N_2_, CO, CO_2_, NO_2_ (1–5 cm/s).^107^ The enzymatic oxidation of H_2_ into protons and electrons can be performed by three different types of hydrogenases ([NiFe], [FeFe], and [Fe]),^108^ but only those that incorporate the nickel ion (EC 1.12.1.2) are soluble proteins able to couple the dihydrogen breakdown with the reduction of NAD(P)^+^ cofactors. Compared to hydrogenases using exclusively iron as a metal ion cofactor, soluble [NiFe] hydrogenases also show greater tolerance to oxygen,^109^ a property that makes them highly versatile enzymes for biocatalysis, as well as synthetic biology.

**Figure 6:**
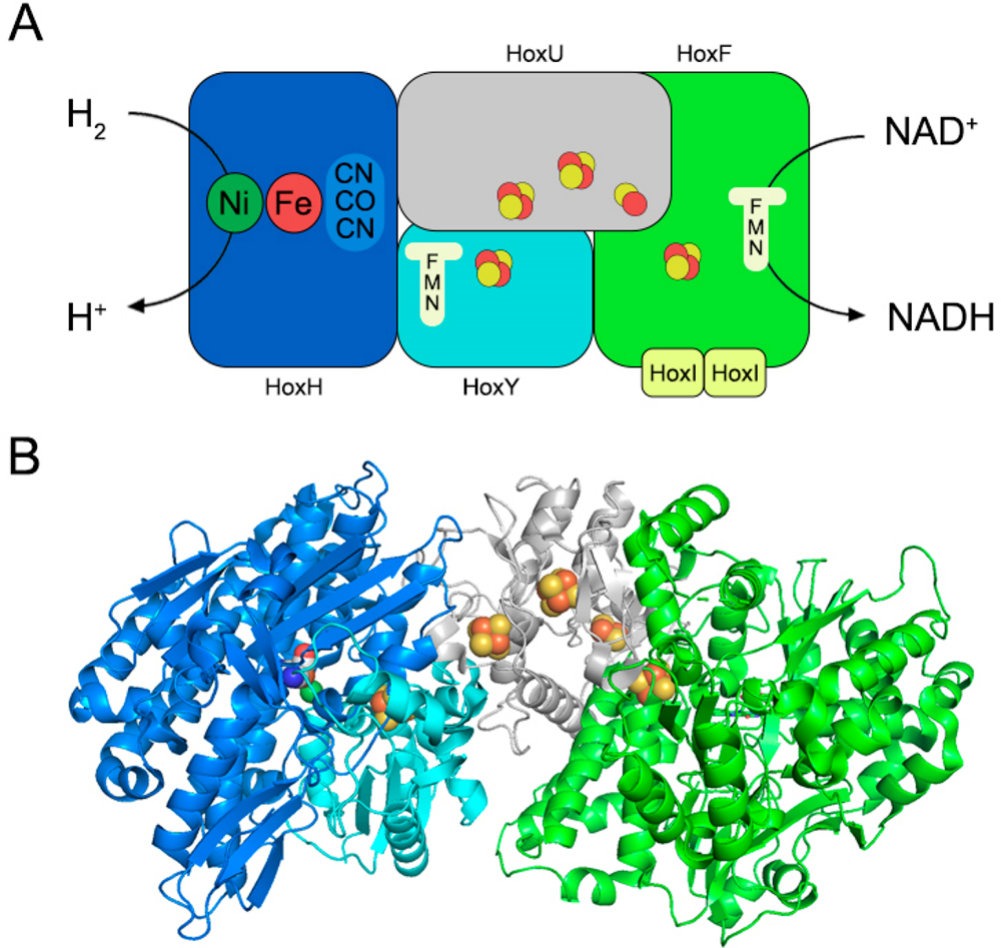
Soluble [NiFe]-hydrogenase structure. (A) The scheme of the subunit and cofactor assembly. The hydrogenase module HoxHY initiates the dihydrogen oxidation (HoxH in blue, HoxY in cyan). Different iron–sulfur clusters (red and yellow balls) allow the electron transfer from the hydrogenase to the diaphorase subunit HoxFU (HoxU in gray, HoxF in green), leading to the NAD^+^ reduction into NADH. The binding of 2 identical HoxI subunits (in yellow) to HoxF generates an hexameric assembly that promotes the reductive-protein activation by NADPH. (B) The tetrameric structure of SH from *Hydrogenophilus thermoluteolus* TH-1 (PDB entry: 5XF9). The color scheme is the same used in A.

Since soluble [NiFe]-hydrogenases (SHs) encompass a diverse group of heteromeric metalloproteins, we will here describe only the most studied homologue from *Cupriavidus necator* H16 (formerly known as *Ralstonia eutropha*)^110^ as a representative model for SHs. As illustrated in Figure 6A, they have a tetrameric active core composed of the hydrogenase (HoxHY) and the NAD(P)^+^-diaphorase (HoxFU) modules, along with two additional identical subunits (HoxI_2_), introducing a further nucleotide-binding site to the protein complex.^76^ The coordination of the metals of the [NiFe] active center, in the HoxH subunit, is mediated by cysteine residues for the nickel ion and by the nonprotein ligands CO and cyanides (CN^–^) for the iron ion. The electron transfer from H_2_ to NAD^+^ is made possible by the further incorporation of 2 molecules of flavin mononucleotide (FMN-a and FMN-b), respectively in HoxY and HoxF, and several iron–sulfur clusters along the tetrameric complex (one [4Fe-4S] in HoxY, two [4Fe-4S] and one [2Fe-2S] in HoxU, one [4Fe-4S] in HoxF). The 2.6 Å tetrameric structure (Figure 6B) of the SH from *Hydrogenophilus thermoluteolus* TH-1 (approximately 40% sequence identity with the SH of *C. necator*) currently offers the most complete model for a mechanistic understanding of the SH family.^111^

In terms of applicability, the kinetic parameters of SHs are favorable, as they show high affinity for both the substrates, as well as a turnover number greater than 100 molecules converted in one second. The pH optimum of SH corresponds to 8.0 and could be maintained in both potassium phosphate (KP_i_) and Tris buffers, although only the tetrameric form is stable in the latter buffer. The ionic strength of the reaction medium is also a crucial parameter for enzymatic functionality, with the protein being inhibited by more than 70% above 200 mM KP_i_.^75^ Importantly, unless anaerobic conditions are ensured thorough the whole isolation procedure, the purified SH requires a short reducing pretreatment (with more than 5 μM NADH) to be active in both the conformations of tetramer or hexamer, as the enzyme is inactivated over time when in contact with the air dioxygen.^76^ A recent insight on the active site of SH of *H. thermoluteolus* suggested a protective role of Glu32 from oxidative damage,^112^ preventing the access of dioxygen to the embedded and cysteine-coordinated nickel ion; further exploration on the mechanism of oxygen tolerance is needed to promote a broader biotechnological use of SHs. In-depth studies on the cofactor specificity have not been conducted so far on SHs, but a first insight was provided by the work of Preissler and colleagues:^113^ they relaxed the strict NAD^+^-dependence of SH isolated from *C. necator* via a double mutation E341A/S342R that allowed to recognize also NADP^+^ as a substrate with similar affinity to NAD^+^, although with a four times lower turnover number.

Similarly to formic acid, also the use of the dihydrogen oxidation to feed reductive reactions would not affect the carbon balance of the metabolism, as only one proton is released into the compartment while NAD(P)H is formed. But unlike a weak acid, H_2_ permeation does not lead to luminal acidification or water efflux, maintaining the original pH and volume at which our synthetic cells would operate their metabolic functions. The advantages that reaction products do not have to be disposed of and dihydrogen is readily membrane permeable are somewhat counterbalanced by the multiplicity of subunits and prosthetic groups required for the functionality of SHs: the protocols for the purification and activity of these dehydrogenases are less trivial than the other dehydrogenases here described, but in any case feasible with the adequate procedures extensively reported elsewhere.^108,114,115^

## Glycerol

**Figure 7:**
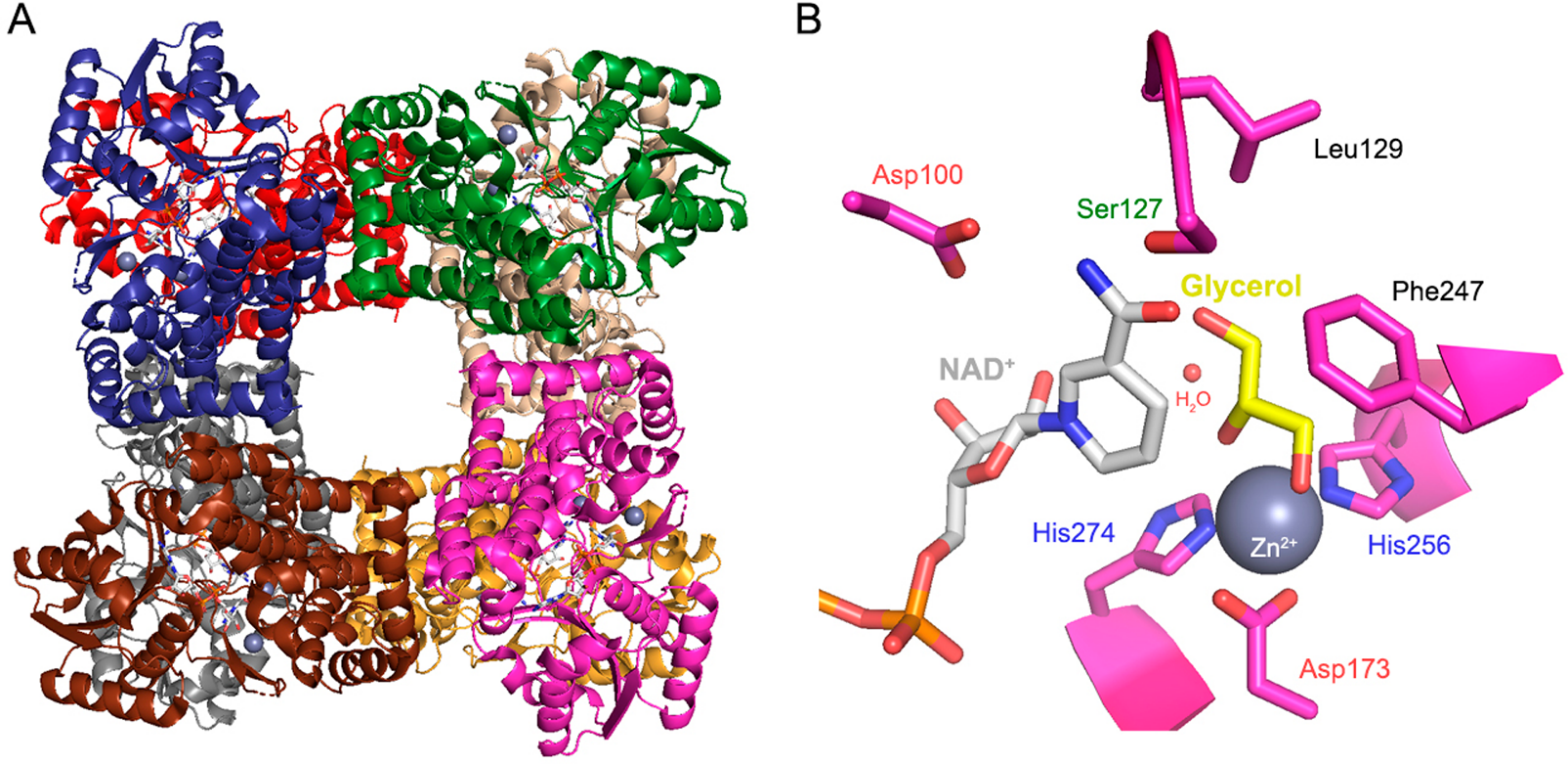
Glycerol dehydrogenase from *Geobacillus stearothermophilus*. (A) The enzyme has an homo-octameric assembly in solution, here illustrated in complex with NAD^+^ (PDB entry: 1JQ5). (B) Overview of the active site of GlyDH in complex with the substrates. By superimposing the structures in complex with NAD^+^ and glycerol (PDB entries: 1JQ5, 1JQA), the essential amino acids to accommodate both substrates are highlighted. Asp100, Ser127, and Leu129 establish a hydrogen bond network with the carboxamide group of the nicotinamide moiety. Glycerol is correctly oriented toward the catalytic center through van der Waals interactions between Phe247 and the carbon atoms of the substrate. The coordination of zinc is mediated by dipole interactions with Asp173, His256, His274, and a water molecule.

Glycerol is a polar and nontoxic triol able to diffuse across phospholipid membranes (*P* = 2 × 10^–5^ cm/s in DOPC).^48^ These characteristics make it a suitable substrate to be exploited for the bottom-up assembly of synthetic cells, as already shown for the synthesis of the phospholipid precursor glycerol-3-phosphate within liposomes fed in a continuous-flow dialysis apparatus.^12^ In addition to the supply of carbon for assimilation, glycerol can also provide reducing equivalents to NAD(P)^+^ cofactors. The NAD^+^-dependent glycerol dehydrogenase (GlyDH, EC 1.1.1.6) catalyzes the oxidation of glycerol into dihydroxyacetone, and the enzyme can also oxidize glycerol-derivatives albeit at a significantly lower rate.^78^ The recognition of a broad range of substrates is a well-known characteristic of the heterogeneous group of polyol dehydrogenases: the same applies to GlyDH, considered as the representative model of the family III of polyol dehydrogenases.^59^ Biochemical understanding on GlyDHs is based on the studies carried out on the protein isolated from *Geobacillus stearothermophilus*.^116^ Its kinetic parameters are strongly affected by the pH of the reaction medium, revealing better *K*_M_ and *k*_cat_ values at alkaline pH. Inactivation by chelating agents provided the first evidence on a strict dependency of GlyDH on zinc (Zn^2+^), later proved to be embedded in the active site of the protein. In fact, crystallographic structures in the apo- and holo-forms^59^ contributed to shed light not only on the oligomeric state (Figure 7A), but also on the mechanistic basis behind the metal coordination and the catalytic action of the enzyme (Figure 7B). Three specific amino acids (Asp173, His256, and His274) and a water molecule allow the tetrahedral coordination of Zn^2+^ to the protein active site. Other key amino acids correctly orient the substrates forming hydrogen bonds with the nicotinamide ring and van der Waals interactions with glycerol. Studies on the cofactor specificity for GlyDH are not available but, looking at its holo-structure in the presence of NAD^+^ (similarly to what observed in nonmetal FDHs with Asp221), an aspartate in position 39 lies in the proximity of the 2′-OH group of the adenine ribose forming a hydrogen bond with the coenzyme: the mutagenesis of Asp39 (and neighboring amino acids) could therefore be investigated to develop a NADP^+^-dependent GlyDH.

The choice of glycerol to feed synthetic cells with reducing equivalents can be beneficial on multiple levels. In addition to bypassing the need for membrane proteins, it can increase the operational stability of the compartmentalized metabolism: glycerol is a stabilizing agent for numerous proteins, allowing to prolong the enzymatic functionality over time.^117^ While the glycerol that does not react stoichiometrically with the cofactors can boost metabolic stability, the reaction product dihydroxyacetone can potentially permeate across phospholipid membranes without the support of a protein translocator. Recent studies on bacterial and archaeal lipid membranes have in fact highlighted membrane permeability comparable to both glycerol and dihydroxyacetone,^118^ suggesting not too significant differences between their permeability rates. The luminal entry of glycerol, followed by the diffusion out of its oxidation product, maintains unaltered the carbon mass of the reactants enclosed in the vesicles, resulting in the orthogonal provision of reducing equivalents to downstream NAD(P)H-dependent reactions.

## Enzymatic Transhydrogenation: A Carbon-Free Approach to Tackle the Cofactor Specificity Bottleneck

**Figure 8:**
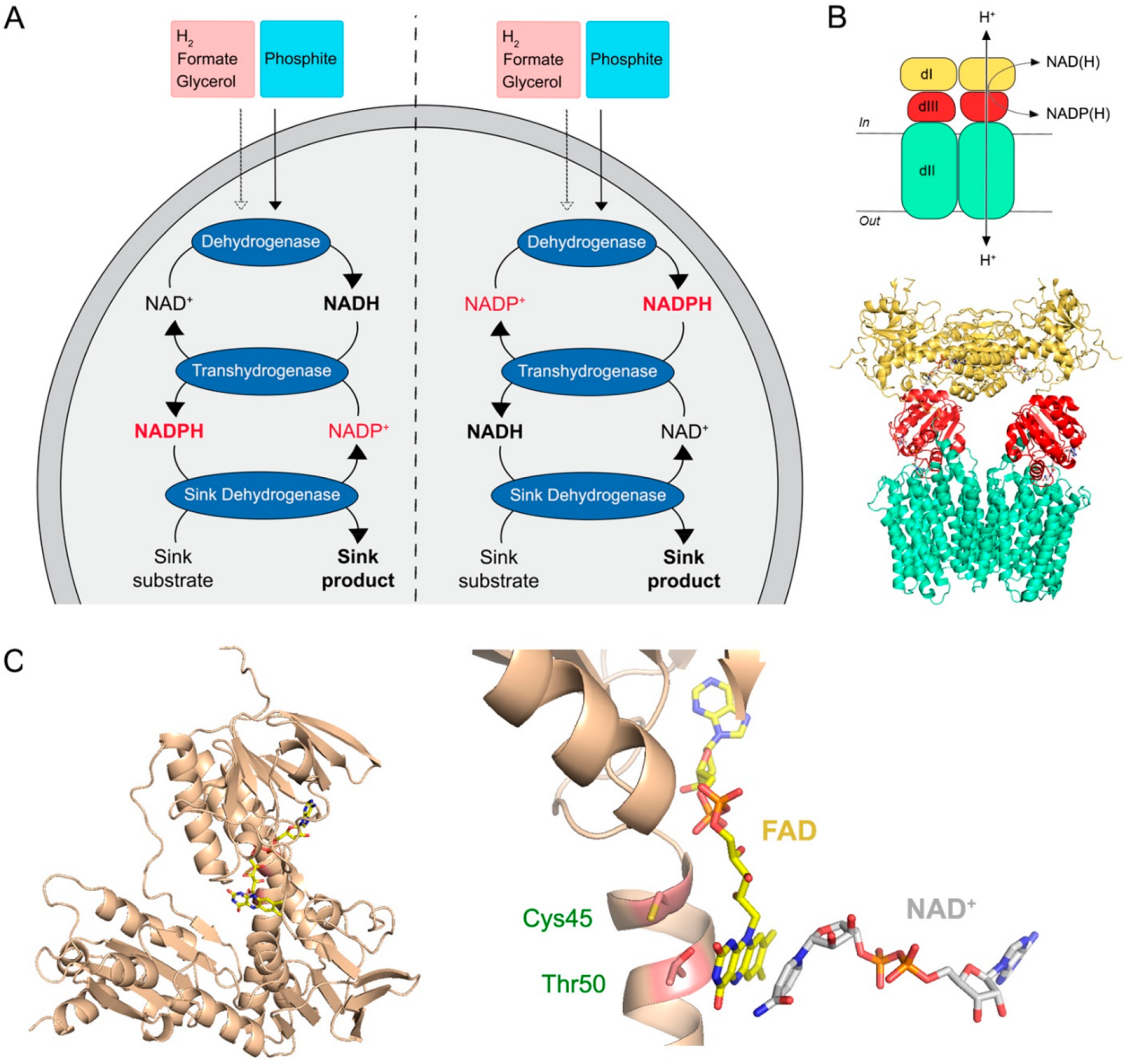
Transhydrogenases in synthetic cells. (A) Scheme of the redox regeneration. Following the luminal access of the electron donor (passively indicated by the dashed arrow, actively by the solid arrow), NAD^+^ (left side) or NADP^+^ (right side) is reduced by an upstream specific dehydrogenase. A downstream transhydrogenase transfers electrons from one cofactor to another, overcoming the limitation of cofactor specificity, and reoxidizing the cosubstrate NAD(P)H during transhydrogenation. An additional sink enzymatic reaction can take advantage of the new reduced cofactor, while pulling the transhydrogenase reaction out of equilibrium. (B) Structural organization of membrane transhydrogenases. On top, the scheme of the protein assembly. All known mTHs are organized into three distinct domains: dI, NAD(H)-binding in yellow; dII, transmembrane in cyan; dIII, NADP(H)-binding in red. On bottom, the heteromeric structure of the holo-enzyme NNT from *Ovis aries* mitochondria (PDB entry: 6QTI) in complex with NAD^+^ and NADP^+^. (C) Structural and catalytic insight of soluble transhydrogenases. On the left, the monomeric predicted structure of the soluble transhydrogenase from *E. coli* K-12 (AlphaFold entry: AF-P27306) embedding the prosthetic group—FAD—upon superimposition with the related lipoamide dehydrogenase from *Thermus thermophilus* HB8 (PDB entry: 2EQ7). On the right is a zoom into the FAD-binding domain in complex with NAD^+^, highlighting cysteine 45 and threonine 50, as they are conserved amino acids in sTHs for efficient catalytic activity.

While NAD(H) is mostly linked to energy provision in combination with ATP generation during the catabolic breakdown of high-energy compounds,^119^ NAD(P)H plays a main role in biosynthetic and antioxidant pathways.^120^ The overview of suitable dehydrogenases presented above showcases how cofactor specificity represents a key feature of redox enzymes. Multiple structural studies combined with protein engineering efforts have been contributing to overcome this limitation by changing or loosening the cofactor preference of specific oxidoreductases,^80,121−123^ but some dehydrogenases (SHs, GlyDHs) are still not extensively characterized with respect to cofactor specificity. More importantly, even when it is possible to reduce both coenzymes through the action of native or mutant (PDHs, FDHs, SHs) enzymes, it is likely that the demand for two reduced cofactors will not be constant over the lifetime of a synthetic cell, thus the ability of transferring reducing equivalents between the two compounds is desirable. Specific pyridine nucleotide transhydrogenases (THs) would allow to transfer the reducing equivalents from one reduced cofactor to another oxidized one, cycling the redox status of the nicotinamides and therefore to link the different pathways of the reductive metabolism dependent on both NADH and NADPH (Figure 8A). THs exist as membrane (mTHs) or soluble (sTHs) enzymes catalyzing the reversible hydride transfer among cofactors, although with very different mechanisms.^124^ Their integration in a minimal metabolism may provide a useful alternative to overcome the barrier imposed by cofactor specificity, while preventing the requirement for extra metabolic modules to regenerate the redox status of both nicotinamide cofactors via formation of new metabolites (mostly carbon-based) that should then be internally recycled or exported outside of the vesicular lumen. We now proceed to briefly describe the main biochemical and structural features of both types of THs characterized to date.

## Membrane Transhydrogenases

The membrane transhydrogenase family (TCDB 3.D.2, also known as proton-translocating transhydrogenase family) includes proteins partially embedded in the phospholipid bilayer that protrude a catalytically active domain into the cytosol (in bacteria) or the mitochondrial matrix (in eukarya) to catalyze transhydrogenation. The hydride transfer from NAD(P)H to NAD(P)^+^ is coupled to the translocation of a proton across the membrane (Figure 8B). *In vivo*, the normal ratios of NADH/NAD^+^ and NADP^+^/NADPH favor the transhydrogenation toward the formation of NADPH, which is then released from the dIII domain into the cytosol by importing a proton (available from the physiological electrochemical gradient).^125^ Nonetheless, the reverse reaction (NADH-forming) can also be mediated by mTHs exporting a proton, as demonstrated *in vitr*o in submitochondrial particles.^126^ The overall structural organization of mTHs is strongly conserved, although they can consist of 1 to 3 polypeptide chains (e.g., 1 in mammalian mitochondria, 2 in *E. coli*, 3 in *Thermus thermophilus*).^127^ The mTHs are natively homodimers; each protomer can be divided into 3 distinct domains (Figure 8B): the most membrane-distal dI (specific for NAD(H)-binding), the central transmembrane site dII, and the globular domain most proximal to the membrane dIII (binding NADP(H)). Structural studies on the mammalian and *T. thermophilus* mTHs proposed a common “antiphase” catalytic mechanism (also described as “division of labor”)^128,129^ in which the two dIII protomers alternate opposite “face-up” and “face-down” conformations: the “face-up” conformation is needed for hydride transfer, while the “face-down” conformation allows proton translocation across the membrane.

Biochemically, the best characterized mTHs are NNT from *Bos taurus* mitochondria and PntAB from *E. coli*.^130,131^ Upon purification, they both have similar high affinities for oxidized and reduced nicotinamide cofactors (*K*_M_ values between 2 and 150 μM) and comparable turnover numbers around 10–15 s^–1^. The mTH-mediated transhydrogenation seems to be easily integrated with further essential biological processes *in vitro*: the recent work by Graf et al.^131^ demonstrated how purified PntAB can work synergistically with ATP synthase, driving the synthesis of ATP through the generation of a proton motive force at the expense of the transhydrogenation from NADPH to NAD^+^. Nonetheless, the activity of PntAB in proteoliposomes mostly takes place—and is thus measured—extraluminally as it assumes a preferential orientation (∼75%) with the NAD(P(H))-binding domains extruding from the outer surface of the vesicles. Therefore, methods that promote correct orientation to ensure transhydrogenation in the vesicular lumen are desirable for efficient utilization of mTHs in synthetic cells. Alternatively, strategies to remove traces of external protein activity could be applied (e.g., enzymatic scavenger systems^14^).

## Soluble Transhydrogenases

The sTHs (EC 1.6.1.1) are energy-independent flavoproteins that tightly bind FAD as prosthetic group to perform transhydrogenation between nicotinamide cofactors.^124^ Although they are mainly associated with the maintenance of the NADPH/NADP^+^ pool by reoxidizing the surplus of cytosolic NADPH,^132^ sTHs can reversibly mediate transhydrogenation both *in vivo* and *in vitro*.

The most studied sTHs belong to *Pseudomonas fluorescens*, *Azotobacter vinelandii* and *E. coli* (each of them annotated as SthA), with the first 2 orthologs displaying high sequence identity (85%) with each other; SthA from *E. coli* is instead more evolutionarily distant from the other 2 sTHs, with a degree of identity that does not exceed 60% and 64%, respectively. Interestingly, despite the homology the proteins have very different oligomeric conformations. Electron microscopy studies have shown filaments up to 300 nm in length for SthA from *A. vinelandii*, and nearly one micrometer long for SthA from *P. fluorescens*, the functional unit of which is assumed to be an octamer.^133,134^ In *E. coli*, SthA adopts a globular conformation characterized by 8 protomers with an overall radius around 8 nm.^135^ High resolution structures of the octameric complex have not yet been resolved for any sTH homologue, but the predicted monomer structure (Figure 8C, left panel) is available on the AlphaFold database.

Assuming the size of metabolically active synthetic cells in a range between 1 and 10 μm in diameter,^42^ we consider the homo-octameric SthA from *E. coli* more suitable than the filamentous ones to guarantee the shuttle of hydrides between cofactors in a compartmentalized metabolism; in this way it would be possible to minimize the effects of excluded volume, and consequently preserve useful luminal space to encapsulate further metabolic modules.^51^ We therefore focus on the biochemical features of SthA from *E. coli* as a representative model for the biochemistry of sTHs.

The purified SthA catalyzes the concomitant oxidation of NADPH and reduction of the non-natural substrate thio-NAD^+^ with a turnover number between 170 and 260 s^–1^ and *K*_M_ values around one hundred micromolar for both substrates.^136^ The SthA-mediated reaction consuming NADH while reducing thioNADP^+^ has also been recently characterized, revealing a lower *k*_cat_ value of 10–15 s^–1^, and higher affinity for thioNADP^+^ compared to NADH (0.1 and 2.6 mM, respectively).^135^ Substrate inhibition occurs in both directions of reaction with NADPH, NADH and thioNADP^+^, as well as high concentrations of phosphate ion decrease the activity of SthA to 15–25% compared to a phosphate-free reaction medium. The enzyme works optimally at pH 7.5–8.5 and at a temperature of 35 °C, maintaining full activity for at least 3 weeks when incubated at 4 °C.

sTHs are related to the disulfide reductase family of flavoproteins, and retain an exceptional CXXXXT domain (instead of the typical CXXXXC domain for reducing disulfides) which promotes tight FAD-embedding to the active site (Figure 8C, right panel) and allows for efficient transhydrogenation.^135^ As reported for several flavoproteins, SthA also exhibits oxidase activity both in the absence of an oxidized cofactor as acceptor and during transhydrogenation. This reactivity with dioxygen leads to a marginal formation of hydrogen peroxide and superoxide anion (together they intercept 2% of reducing equivalents from NADH to thioNADP^+^, while the remaining 98% is used for transhydrogenation). Although small, the formation of such side products should be taken into account in the design of a “in confinement” enzymatic network, as they could react and eventually damage membranes, (deoxy)ribonucleic acids, and other enzymes.^137^ The addition of antioxidant systems such as glutathione reductase/peroxidase, catalase, or superoxide dismutase would overcome the problem by forming harmless molecules such as reduced glutathione or water.

## Conclusions

The pivotal role of NAD(H) and NADP(H) for redox metabolism requires strategies to reduce the cofactors by external electron donors within artificial cells to efficiently feed any other reductive reaction in a controllable manner. By establishing parameters for the reduction of nicotinamides enclosed by selectively permeable vesicles, we proposed four possible candidates as suitable electron donors. Our analysis employed an “enzymatic” view (dehydrogenases and membrane transporters) by reviewing the structural and biochemical properties of the protein biocatalysts transporting or oxidizing these compounds for the downstream reduction of NAD(P)^+^ cofactors. Considering proteins as the crucial catalysts for the functionality of metabolic networks, keeping a system with life-like properties out of thermodynamic equilibrium, we advocate for an in-depth understanding of the chosen biocatalyst(s) for robust activity in minimal synthetic cells.

From our work it emerges that each of the considered redox donors exhibits attractive properties in some respects, but certain limitations in others. Phosphite demands a membrane transporter for uptake (and the product phosphate requires a dedicated export system) and it suffers from a lack of purified proteins with available characterization. Membrane-permeable donors show different side effects: luminal acidification in the case of formate permeation, relative oxygen sensitivity for the enzyme(s) utilizing molecular hydrogen, relatively slow diffusion in and out the compartment for glycerol and its oxidized product (dihydroxyacetone). For enzymes in particular, it is also important to consider whether they need to incorporate one or more prosthetic groups to perform their function. An enzyme embedding multiple metal ions (e.g., [NiFe]-hydrogenase) is less trivial to work with than metal-free proteins, and much less versatile during the steps of protein purification, vesicular encapsulation, and *in vitro* activity. More importantly, the cofactor specificity of dehydrogenases appears as a recurring element that changes and at the same time distinguishes each specific homologue. Transhydrogenases are solutions to overcome this issue through the hydride transfer between NAD(H) and NADP(H), with the benefit of avoiding the direct involvement of other carbon-based metabolites.

Likely many efforts focused on constructing synthetic cells will include the establishment of NADPH-dependent biosynthetic pathways, requiring a sufficiently high ratio of NADPH/NADP^+^ to push their reactions. As many available donor systems primarily generate NADH, the use of transhydrogenases is a promising way of conveying the reducing power from one cofactor to another. The membrane-bound transhydrogenases are prime candidates to convert NADH into NADPH at the expense of proton translocation across the membrane. The energy investment will help to overcome the thermodynamic gap of transferring a hydride from NADH, upward to maintaining a high NADPH/NADP^+^ ratio to push biosynthesis. This proton motive force can be provided for example by ATP synthase or proton-pumping rhodopsin, as already demonstrated in liposome systems.^13^

For thermodynamically very favorable electron donating substrates (e.g., phosphite, formate, and hydrogen), the NADH/NAD^+^ ratio that can be generated by the electron donor may be much higher than the typical ratio found in most bacteria (0.03–0.32).^22^ In this case, a non-energy-consuming soluble transhydrogenase may be sufficient to drive the hydride transfer from NADH to NADP^+^: SthA from *E. coli* has been employed recently for this purpose in synthetic cells in synergy with formate as electron donating substrate and glutathione as electron sink.^14^

To conclude, the selection of an electron donor should be made on the basis of the experimental conditions of the metabolic processes to be integrated in cell-like systems, tailoring the choice of the most suitable protein homologue so that it can function in optimal conditions, without interfering with other encapsulated components. In this regard, protein engineering is a fruitful resource available to the scientific community to broaden the range of scenarios in which a protein can be used (different optimum of pH and temperature, stability in selected buffers, better kinetics, substrate specificity). With further studies to elucidate the structure, and therefore the biochemistry of more and more enzymes, we will be able to customize their utilization in support of bottom-up synthetic biology.

Our recently demonstrated redox regeneration system based on formic acid may serve as a blueprint to develop redox homeostasis for synthetic cells. This system, or alternative systems, following the guidelines provided in this work, can support the further construction of more complex metabolisms in bottom-up synthetic cells.

## Abbreviations

NAD^+^: oxidized nicotinamide adenine dinucleotide
NADH: reduced nicotinamide adenine dinucleotide
NADP^+^: oxidized nicotinamide adenine dinucleotide phosphate
NADPH: reduced nicotinamide adenine dinucleotide phosphate
ATP: adenosine triphosphate
*E*°′: standard potential reduction
CO: carbon monoxide
CO_2_: carbon dioxide
H_2_: molecular hydrogen
H^+^: hydrogen ion
FAD: oxidized flavin adenine dinucleotide
HCOO^–^: formate or anionic form of formic acid
DOPC: 1,2-dioleoyl-*sn*-glycero-3-phosphocholine
EC: enzyme commission
GDH: d-glucose dehydrogenase
SO_4_^2–^: sulfate
HSO_3_^2–^: sulfite
CODH: carbon monoxide dehydrogenase
ABC-transporter: ATP-binding cassette transporter
ADP: adenosine diphosphate
Na^+^: sodium ion
TX-TL: transcription–translation
NiFe: nickel–iron
*P*: permeability coefficient
PO_4_^3–^: phosphate
HPO_3_^2–^: phosphite
*K*_eq_: equilibrium constant
PDH: phosphite dehydrogenase
*k*_cat_: turnover number
*K*_M_: Michaelis–Menten constant
PDB: Protein Data Bank
*K*_D_: dissociation constant
TCDB: transporter classification database
FDH: formate dehydrogenase
Mo: molybdenum
W: tungsten
N3: azide
Fe: iron
O_2_: dioxygen
N_2_: nitrogen gas
NO_2_: nitrite
SH: soluble [NiFe]-hydrogenase
CN^–^: cyanide
FMN: flavin mononucleotide
KP_i_: potassium phosphate
Tris: tris(hydroxymethyl)aminomethane
GlyDH: glycerol dehydrogenase
Zn^2+^: zinc ion
TH: pyridine nucleotide transhydrogenase
mTH: membrane transhydrogenase
sTH: soluble transhydrogenase.
